# Brain cancer mortality in an agricultural and a metropolitan region of Rio de Janeiro, Brazil: a population-based, age-period-cohort study, 1996–2010

**DOI:** 10.1186/1471-2407-14-320

**Published:** 2014-05-06

**Authors:** Adalberto Luiz Miranda Filho, Rosalina Jorge Koifman, Sergio Koifman, Torres Rego Gina Monteiro

**Affiliations:** 1 Environmental and Public Health Program, National School of Public Health, Oswaldo Cruz Foundation, Rio de Janeiro, Brazil; 2 Department of Epidemiology and Quantitative Methods, National School of Public Health, Oswaldo Cruz Foundation, Rio de Janeiro, Brazil

**Keywords:** Brain cancer, Age-period-cohort, Agriculture, Trend, Pesticide

## Abstract

**Background:**

Individuals who live in rural areas are at greater risk for brain cancer, and pesticide exposure may contribute to this increased risk. The aims of this research were to analyze the mortality trends and to estimate the age-period-cohort effects on mortality rates from brain cancer in two regions in Rio de Janeiro, Brazil.

**Methods:**

This descriptive study examined brain cancer mortality patterns in individuals of both sexes, >19 years of age, who died between 1996 and 2010. They were residents of a rural (Serrana) or a non-rural (Metropolitan) area of Rio de Janeiro, Brazil. We estimated mortality trends using Joinpoint Regression analysis. Age-period-cohort models were estimated using Poisson regression analysis.

**Results:**

The estimated annual percentage change in mortality caused by brain cancer was 3.8% in the Serrana Region (95% confidence interval (CI): 0.8–5.6) and -0.2% (95% CI: -1.2–0.7) in the Metropolitan Region. The results indicated that the relative risk was higher in the rural region for the more recent birth cohorts (1954 and later). Compared with the reference birth cohort (1945–49, Serrana Region), the relative risk was four times higher for individuals born between 1985 and 1989.

**Conclusions:**

The results of this study indicate that there is an increasing trend in brain cancer mortality rates in the rural Serrana Region in Brazil. A cohort effect occurred in the birth cohorts born in this rural area after 1954. At the ecological level, different environmental factors, especially the use of pesticides, may explain regional disparities in the mortality patterns from brain cancers.

## Background

Malignant brain neoplasms are intracranial tumors that occur more frequently in adult males. Approximately 70% of these highly lethal tumors originate in glial cells (gliomas). Only 3% of patients with this histological type of cancer survive for more than 5 years after diagnosis [1-3]. The etiology of brain cancer is not well understood. Genetic and environmental factors contribute to the development of brain cancer [4-6]. Individuals with agricultural occupations and non-farmers living in rural communities have higher mortality rates for some specific cancers, including brain cancer. The main hypothesis presented in the literature that accounts for this excessive mortality is exposure to pesticides [7-11].

The Serrana Region is the main agricultural area in the state of Rio de Janeiro, Brazil, especially for the production of fruits, vegetables, and flowers. This region has the largest per capita consumption of pesticides and fertilizers and the largest numbers of inhabitants engaged in agricultural activities. In contrast, the Metropolitan Region has the lowest per capita consumption of pesticides and fertilizers and the lowest numbers of inhabitants engaged in agricultural activities. These differences in pesticide and agricultural exposure motivated the development of this ecological investigation [12,13].

Because there is no local population-based cancer registry in the state of Rio de Janeiro, this brain tumor mortality study represented an initial approach to examining the magnitude of this health problem. An evaluation of the effects of age, time period, and birth cohort on brain cancer mortality may assist in the ecological-level identification of etiologic factors related to the development of these neoplasms [14]. This approach assumed, a priori, that the effects of age could represent biological changes that occur during aging. The period when death occurs may also reflect important changes in factors that affect mortality (e.g., introduction of new treatments). The cohort effect may indicate changes in exposures that are particular to specific generations [15,16].

The country of Brazil is one of the major consumers of pesticides worldwide, but few studies that evaluate the impact of these substances on population health have been published [17]. The exposure scenario for our study consisted of an agricultural region where workers were given personal protective equipment, but there was little knowledge about the need to use this equipment while at work [18]. In this sense, studies of the health effects of pesticide exposure in agricultural production areas in Brazil might be qualitatively and quantitatively different from studies performed in developed countries. Therefore, this study contributes to the understanding of the brain cancer patterns in areas of intensive pesticide use and explored the environmental hypotheses in the Brazilian context.

The aim of this study was to analyze mortality trends and to assess the age, birth period, and cohort effects on brain cancer mortality rates in the Serrana Region of the state of Rio de Janeiro, and to compare them with rates in the Metropolitan Region of the same state.

## Methods

### Study design and population

This was an ecological study on the distribution of deaths from brain cancer classified as C71 (malignant neoplasm of brain) in ICD-10 [19]. The study population consisted of individuals between 20 and 79 years old living in the Serrana Region and in the Metropolitan Region of the state of Rio de Janeiro between 1996 and 2010. Mortality data were obtained from the database of the Brazilian national Mortality Information System, Ministry of Health [20]. Data on the number of inhabitants during the same period were obtained electronically from the Brazilian Institute of Geography and Statistics (Rio de Janeiro) [21].

### Study area

The Serrana Region of the state of Rio de Janeiro consists of seven municipalities. In 2010, the population size of this region was approximately 710,000 inhabitants. Approximately 90% of the population is distributed among the municipalities of Nova Friburgo and Teresópolis, and the city of Petrópolis [21]. The Serrana Region is the main agricultural area in the state. The 2006 agricultural census reported that 5.34% of the regions’ workers were engaged in agricultural activities [22].

The Metropolitan Region of the state of Rio de Janeiro consists of 19 municipalities, including the capital (Rio de Janeiro). In 2010, 54% of the 11,600,000 individuals that lived in this region resided in the capital city [21]. The 2006 agricultural census reported that 0.01% of workers in the Metropolitan Region were engaged in agricultural activities [22].

### Study variables

Brain cancer mortality rates for each age group were calculated per 100,000 inhabitants and were adjusted by world standard population [23]. We included the variables age (in 5-year intervals), number of deaths (grouped into 5-year periods), the population at risk in the middle of each 5-year interval (person-time), and the study period grouped in 5-year categories in the analysis of age, period, and cohort effects.

### Statistical analysis

We performed a descriptive analysis of mortality rates (means and standard deviations), and of global and specific adjusted rate ratios, by age group.

Trend analysis was performed using log-linear Poisson regression. The objective of this analysis was to identify significant changes in rate patterns during the study period. An estimated annual percentage change (EAPC) was calculated for each change. Results with a p-value <0.05 were considered to be statistically significant. The choice of the model was determined using a permutation method [24]. These analyses were performed using Joinpoint version 3.4 software (Statistical Research and Applications Branch, National Cancer Institute, USA).

During the analysis of the age, period, and cohort effects, and the estimation of values for relative risk (RR), models were adjusted using log-linear Poisson regression modeling. The model assumed that the number of deaths observed during the study period followed a Poisson distribution with constant mortality rates and events that were independent from each other. The logarithm (log) of the mortality rates was an additive function of the parameters as described by

$$\log\left(r_{\mathrm{ij}}\right)=\left({\mathrm{Di}}_{j}/{\mathrm{Pi}}_{j}\right)=µ+\left(A\right)\alpha i+\left(P\right)\beta j+\left(P-A\right)\gamma k$$

where (*r*_*ij*_) = mortality rate expected; Di_j_ = number of deaths in the i-th age group in the j-th period; Pi_j_ = population in the i-th age group and j-th period; A = age, P = period; μ = intercept adjusted mean, αi = effect of the i-th age group; β_j_ = effect of the j-th period; γk = effect of the k-th cohort [25,26]. The model that best fit the data was selected using the deviance function and was assessed by comparing the effects of each parameter in relation to the full model (age, period, and cohort). Models with a p-value <0.05 were considered to be statistically significant.

We chose the parameterization method proposed by Holford [27] to overcome the uncertainty associated with nonidentifiability. The reference group for the age effect was the 20–24 year age group, and the reference for the period effect was the 1996–2000 period. The reference for the generation of births was the median value, because central cohorts are more stable [27,28]. The periods 1945–1949 and 1940–1944 were used for the Serrana and Metropolitan regions, respectively. The statistical software R version 2.15.1, Epi version 1.1.9 (R Foundation for Statistical Computing, Vienna, Austria; http://www.r-project.org) was used for this analysis.

## Results

Between 1996 and 2010, there were 412 deaths caused by brain cancer in individuals >19 years of age in the Serrana Region (mean rate = 4.20 deaths per 100,000 inhabitants; standard deviation = 0.85). There were 5,322 brain cancer deaths (mean rate = 3.39 deaths per 100,000 inhabitants; standard deviation = 0.23) during the same time period in the Metropolitan Region. The mean ages at death were 64 and 65 years in the Serrana and Metropolitan regions, respectively. Compared with the Metropolitan Region, the ratio of adjusted mortality rates in the Serrana Region was higher in all age groups, with a mean increase that was 40% higher.

**Figure 1:**
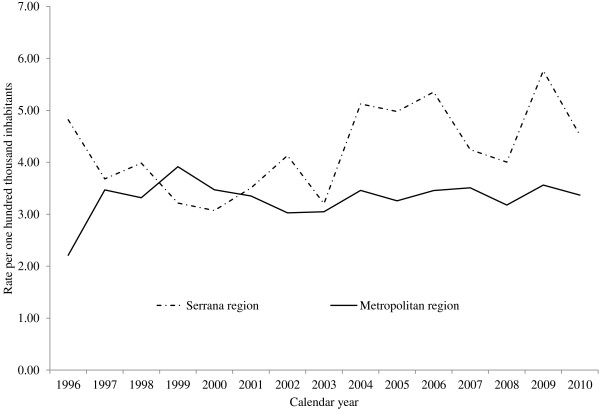
**Trends in mortality from brain cancer adjusted by world standard population in the Serrana region and Metropolitan area of Rio de Janeiro between 1996 and 2010.** Axis Y shows the mortality rates per one hundred thousand inhabitants and axis X shows the calendar year.

Figure 1 presents the results for the variation in adjusted mortality rates in the two regions between 1996 and 2010. There were two distinct periods of rate behavior. In the Serrana Region, the EAPC was -9.6% (95% CI: -30.4–17.5) between 1996 and 1999 and was 4.2% (95% CI: 0.4–8.1) between 1999 and 2010. In contrast, in the Metropolitan Region the EAPC was 18.4% (95% CI: -8.8–53.6) between 1996 and 1998 and was -0.5% (95% CI: -1.8–0.9) between 1998 and 2010.

The risk of death from brain cancer increased with age in both regions (Table 1). The greatest increases were in the Serrana Region. The RR for the oldest age group (75–79 years) was 33.63 (95% CI: 15.24–74.22) in the Serrana Region and was 23.78 (95% CI: 22.55–25.07) in the Metropolitan Region (reference age group, 20–24 years).

**Table 1:** Estimates of Relative Risk (RR) and confidence interval with 95% reliability of age, birth cohort and period, in the Metropolitan and the Serrana regions of Rio de Janeiro

**Variables**	**Serrana region**			**Metropolitan region**		
	***N***	**RR**	**95% CI**	***N***	**RR**	**95% CI**
Age						
20 to 24	10	Reference		126	Reference	
25 to 29	9	0.49	0.28 - 0.88	139	1.13	1.09 - 1.18
30 to 34	15	0.64	0.34 - 1.20	207	1.73	1.65 - 1.81
35 to 39	19	1.05	0.52 - 2.11	240	2.07	1.97 - 2.17
40 to 44	27	1.95	0.95 - 4.00	353	3.15	3.00 - 3.31
45 to 49	28	2.51	1.20 - 5.25	464	4.45	4.23 - 4.68
50 to 54	44	6.58	3.08 - 14.08	588	6.47	6.15 - 6.81
55 to 59	46	10.37	4.79 - 22.44	615	8.25	7.84 - 8.69
60 to 64	61	18.87	8.67 - 41.07	719	11.96	11.36 - 12.60
65 to 69	63	27.7	12.65 - 60.66	695	14.99	14.23 - 15.80
70 to 74	49	27.13	12.33 - 59.72	627	17.60	16.70 - 18.54
75 to 79	41	33.63	15.24 - 74.22	549	23.78	22.55 - 25.07
Birth cohort						
1920-24	8	0.58	0.44 - 0.78	124	0.88	0.86 - 0.90
1925-29	31	0.80	0.67 - 0.94	374	1.03	1.02 - 1.05
1930-34	55	0.70	0.61 - 0.80	646	0.94	0.94 - 0.95
1935-39	48	0.59	0.52 - 0.52	685	0.99	0.98 - 1.00
1940-44	52	0.68	0.61 - 0.75	648	Reference	
1945-49	58	Reference		625	1.04	1.03 - 1.05
1950-54	35	0.81	0.72 - 0.93	603	1.06	1.04 - 1.07
1955-59	34	1.19	1.01 - 1.41	497	0.97	0.95 - 0.98
1960-64	32	2.00	1.53 - 2.60	370	0.93	0.91 - 0.94
1965-69	21	1.67	1.20 - 2.32	265	0.90	0.88 - 0.92
1970-74	14	2.23	1.46 - 3.42	195	0.92	0.89 - 0.95
1975-79	12	2.72	1.54 - 4.81	152	0.86	0.83 - 0.89
1980-84	7	4.07	1.83 - 9.04	96	0.90	0.86 - 0.95
1985-89	5	4.17	1.79 - 9.74	42	0.95	0.89 - 1.02
Period						
1996 to 2000	102	Reference		1554	Reference	
2001 to 2005	129	0.98	0.92 - 1.03	1718	0.98	0.97 - 0.98
2006 to 2010	181	1	1	2050	1	1

The median birth cohort was the 1945–1949 period for the Serrana Region. The RR was positive, and statistically significant, from 1955–1959, and was 4.17 (95% CI: 1.79–9.74) for the youngest individuals, born between 1985 and 1989. In the Metropolitan Region, the median occurred between 1940–1944 and the RR ranged from 0.89 (95% CI: 0.83–0.89) to 1.03 (95% CI: 1.04–1.07).

**Figure 2:**
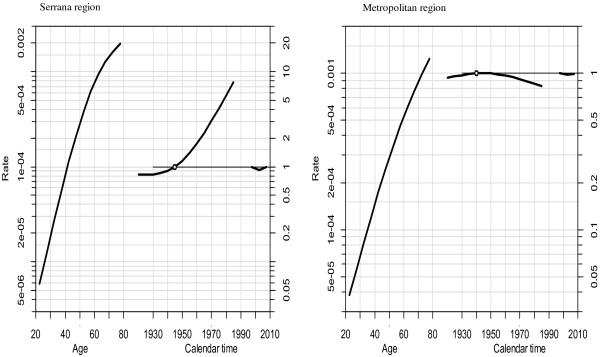
**Estimates of the age-period-cohort effects on brain cancer mortality in residents of the Serrana and Metropolitan regions of the state of Rio de Janeiro, 20 to 79 years of age, from 1996 to 2010: The figures shows: Right – brain cancer mortality rates to 100 thousand inhabitants; Center - brain cancer mortality effects by birth cohort.** Left – effects by death period.

**Figure 3:**
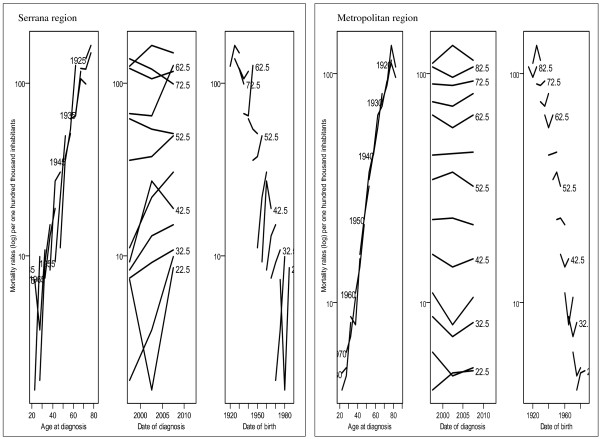
**Comparing age-period-cohort of age-specific mortality rate.** Axis X shows the effect of Age-period-cohort and axis Y shows the (log) mortality rates per one hundred thousand inhabitants.

Figure 2 illustrates the age, period, and cohort effects, and reveals differences between birth cohort effects in the Serrana and Metropolitan regions. Figure 3 presents the results for an age-period-cohort comparison of age-specific mortality rates. Table 2 summarizes the goodness of fit results for the models. The complete model reflects the best fit of the individual effects of age, period, and cohort compared with two factors only.

**Table 2:** Goodness of fit of age-period-cohort models

**Model**	**Serrana region**				**Model**	**Metropolitan region**			
	**Resid. Df**	**Resid. Dev**	**Deviance**	**p-value**		**Resid. Df**	**Resid. Dev**	**Deviance**	**p- value**
Age	409	379.1			Age	5,394	5786.8		
Age-drift	408	341.55	37.551	0.000	Age-drift	5,393	5778.9	7.926	0.005
Age-Cohort	407	313.66	27.896	0.000	Age-Cohort	5,392	5665.6	113.305	0.001
Age-Period-Cohort	406	308.16	5.501	0.019	Age-Period-Cohort	5,391	5569.5	96.103	0.001
Age-Period	407	330.42	-22.267	0.002	Age-Period	5,392	5696.6	-127.108	0.001
Age-drift	408	341.55	-11.129	0.001	Age-drift	5,393	5778.9	-82.301	0.001

## Discussion

The results indicated that there were differences in trend patterns between the two regions. The Serrana Region had higher mortality rates and an increasing trend in mortality over the period analyzed (1996–2010). In contrast, an opposite trend occurred in the Metropolitan area. Mortality rates were lower and declined during the study period, although the decrease was not statistically significant.

Monteiro and Koifman [29] reported an increase in brain cancer mortality rates in Rio de Janeiro between 1980 and 1998 in individuals >65 years of age. Legler et al. [30] analyzed brain cancer mortality rates in the United States between 1975 and 1999, and reported a stable distribution of mortality rates, except in the age group between 64 and 74 years of age. This group had an increase of 5.5% in the EAPC between 1979 and 1995 [30]. In the Umbria Region of Italy, Stracci et al. [31] reported an increasing trend in brain cancer mortality rates of 2.33% (95% CI: 1.42–3.23) in males and 1.78% (95% CI: 0.62–2.95) in females.

The increases in brain cancer incidence and mortality rates that have occurred in recent decades may be attributed to improved diagnostic capability that has resulted from the use of computed tomography (CT) and magnetic resonance imaging (MRI). Population aging has likely also contributed to this change, because age represents an important risk factor for intracranial tumors [32-34]. However, new technologies and aging do not fully explain the increases in incidence and mortality, and there may also be a significant contribution from environmental risk factors [35].

Differences in the magnitude of brain cancer mortality rates observed in this study cannot be explained by greater access to MRI and CT scans. The magnitude of the adjusted mortality rates in the Serrana Region is somewhat higher than the rates in Rio de Janeiro, which has greater access to these diagnostic tools. One hypothesis for the dissimilarity is differences in patterns of exposure to distinct environmental carcinogens between the two regions.

The result of this study indicated that there was a statistically significant age effect on the distribution of brain cancer mortality rates in both regions. Age is an important risk factor in the development of several types of tumors. The number of cell divisions increases during human aging. During cell division errors in DNA replication occur that are critical for the formation of mutations. When these mutations occur in DNA repair mechanisms, they can result in the development of tumors [36]. Flaws in DNA replication can also be induced by specific environmental agents [37].

The most recent birth cohorts in the Serrana Region had higher RRs. This effect may reflect changes in exposures to environmental agents that occurred after 1950, and that have been present since then. Environmental factors likely contribute to the risk of developing brain cancer. Many substances are inducers or promoters of carcinogenesis, including several pesticides [38-41].

The hypothesis for this difference in RR among the birth cohorts of the two regions accounts for differences in patterns of environmental exposures. The greater RRs in the 1980s cohorts may reflect exposures that occurred in childhood, because those individuals were ≤30 years old when they died. Exposure to pesticides in utero and during childhood is a potential risk factor for the development of brain cancer [42,43]. Humans may be exposed to pesticides from several sources, including pesticides present in food and in agricultural and residential areas [44]. The timing of the exposure during development is also important, because specific developmental periods during childhood are more sensitive to the biological effects associated with pesticide exposure [45]. Exposure during these periods may significantly contribute to the risk of development of cancer in adult life, but the causal relationships are not clear.

Compared with the Metropolitan Region, younger patients in rural regions may not have the same level of access to early and accurate diagnosis and effective treatment. Survival rates of rural patients may be lower because of delayed diagnosis and delayed transfer to the more developed cancer hospitals in the cities. Additionally, the results in Table 1 indicated that age is the strongest risk factor. Individuals <35 years in the Serrana Region and <25 years in the Metropolitan Region had the lowest mortality rates. In the Serrana Region, individuals from the most recent birth cohort had four times greater mortality rates, compared with those born in 1945–1949 (referent birth cohort).

Over the past 30 years, the Serrana Region has gone through a process of agricultural modernization [46]. This region is the main agricultural area in Rio de Janeiro, produces mainly vegetables, fruits and flowers, and employs the greatest numbers of workers engaged in agricultural activities in the state [12,22]. According to Brazilian Institute of Geography and Statistics data, large amounts of pesticides are used to grow fruits, vegetables, and flowers. The 1996 volume of pesticides sold in the Serrana Region represented approximately 50% of the total sales volume in the entire state [13].

Consumption of pesticides in Brazil increased from 600 million liters to 850 million liters between 2002 and 2011. The number of commercialized chemicals increased from 468 in 1995 to 600 in 2003. Per hectare consumption of pesticides increased from 3.2 kg to 3.6 kg between 2000 and 2009. In the Serrana Region, pesticide use has been high since 1986, which suggests that the population has been exposed to high levels of these chemicals over the last three decades. Considering the latency period between exposure and cancer diagnosis, it is reasonable to propose that the high use of pesticides in this region could have contributed to increases in the occurrence of diseases related to pesticides, including brain cancer [47-49].

Most of the pesticides used in horticulture, and fruit and flower cultivation are members of the organophosphate and carbamate classes of pesticides. Over the last few years, the carcinogenesis mechanisms associated with chemical induction and promotion of tumors by chemicals has been well-studied. Organophosphate and carbamate pesticides have two possible mechanisms of carcinogenesis. One mechanism is based on genotoxicity (ability to react with DNA) and the other is based on epigenetic mechanisms (changes that alter genetic expression without modifying the DNA sequence) [50]. *In vitro* evidence indicates that organophosphate pesticides induce DNA mutations and methylation. The herbicide paraquat promotes changes in histone acetylation in cell culture [51-53].

Brain cancer in the Serrana Region should be more investigated further. Other studies have found that farmer and resident rural populations have high estimates of risk of death from specific cancers, especially brain cancers [54,55]. Exposure to pesticides may have an important role for the development of brain cancer, as indicated by the mortality rates that were found in our study.

Our results should be interpreted cautiously because ecological studies can be affected by inherent design limitations [56]. A common limitation of studies that use death certificate data is the accuracy of the mortality statistics. However, in a Rio de Janeiro-based study, Monteiro et al. [57] reported an accuracy of 90.1% in the reporting of death from brain cancer. In the Serrana Region, data on deaths from brain cancer had a positive predictive value of 90% [58]. The ratio of the reported deaths in Chapter 18 (Sign Symptoms and Abnormal Findings in Physical Examination and Laboratorial Works) was 4.95% during the study period, and values <6% indicate good record quality [59]. Another study limitation is inherent to uncertainties attributed to the nonidentifiability of the models [15,28]. The three components age, period, and cohort are linear, and it is impossible to simultaneously estimate all three effects in the regression models. We used a method proposed by Holfrold to account for this problem [27].

This original study detected differences in the epidemiological patterns of brain cancer. Internationally accepted variables were used to study the distribution of disease (e.g., the distribution of mortality by age group (age effect), calendar year of death (period effect), and birth year of the deceased (cohort effect). This approach enabled us to generate hypotheses about the contribution of different environmental factors that may explain regional disparities in the distribution of mortality from brain cancer.

This study contributes to the understanding of ecological risk factors for death from brain cancer. The age-period-cohort model proved to be an efficient analytical method and found important differences in mortality patterns that suggest that there were differences in exposure between the two regions. We also found that there was a significant cohort effect, which suggested that residing in an agricultural area during early life increased the risk of mortality. This result supports the hypothesis that environmental exposures are determinants in mortality from brain cancer. Other studies of this population should be prioritized to determine the individual factors that are associated with the development of cancer.

## Conclusions

The results of this study indicated that there was an increasing trend in brain cancer mortality over time among adults living in an agricultural area in the state of Rio de Janeiro. The exploratory data analysis revealed the presence of significant birth cohort effects on the distribution of mortality in 1954 and later. The RR of mortality from brain cancer was four times higher among individuals born between 1980 and 1989, compared with those born in 1945–1949.

## Competing interests

The authors declare that they have no competing interests.

## Authors’ contributions

ALMF, RJK, SK, and GTRM conceived the study and drafted the manuscript. ALMF collected and analyzed the data. ALMF, RJK, SK, and GTRM discussed the results and reviewed the manuscript. All of the authors read and approved the final paper.

## Pre-publication history

The pre-publication history for this paper can be accessed here:

http://www.biomedcentral.com/1471-2407/14/320/prepub
